# Early disc degeneration in radiotherapy-treated childhood brain tumor survivors

**DOI:** 10.1186/s12891-023-06509-4

**Published:** 2023-05-31

**Authors:** Petra Grahn, Tiina Remes, Reetta Kivisaari, Maria H. Suo-Palosaari, Pekka M. Arikoski, Päivi K. T. Koskenkorva, Päivi M. Lähteenmäki, Tuula R. I. Lönnqvist, Marja K. Ojaniemi, Kirsti H. Sirkiä, Anna K. Sutela, Sanna-Maria Toiviainen-Salo, Heikki M. J. Rantala, Arja H. Harila, Jaakko Niinimäki, Jaro Karppinen, Matti Ahonen

**Affiliations:** 1 Department of Pediatric Orthopedics and Traumatology, University of Helsinki and Helsinki University Hospital, Stenbäckinkatu 9, PL 281, 00029 Helsinki, Finland; 2 Department of Pediatrics and Adolescent Medicine, Oulu University Hospital, and Research Unit of Clinical Medicine, University of Oulu, Helsinki, Finland; 3 Department of Child Neurology, New Children’s Hospital, University of Helsinki, Helsinki University Hospital, Helsinki, Finland; 4 Department of Radiology, University of Helsinki and Helsinki University Hospital, Helsinki, Finland; 5 Department of Diagnostic Radiology, Oulu University Hospital and Research Unit of Medical Imaging, Physics, and Technology Medical Research Center Oulu, University of Oulu, Oulu, Finland; 6 Kuopio Pediatric Research Unit, University of Eastern Finland and Kuopio University Hospital, Kuopio, Finland; 7 Department of Clinical Radiology, University of Eastern Finland and Kuopio University Hospital, Kuopio, Finland; 8 Department of Pediatrics and Adolescent Medicine, Turku University Hospital, Turku University, Turku, Finland; 9 Department of Pediatrics and Adolescence, University of Helsinki and Helsinki University Hospital, Helsinki, Finland; 10 Department of Women’s and Children’s Health, Uppsala University, Uppsala, Sweden; 11 Medical Research Center Oulu, Department of Physical and Rehabilitation Medicine, University of Oulu and Oulu University Hospital, Oulu, Finland; 12 Finnish Institute of Occupational Health, Oulu, Finland; 13 Rehabilitation Services of South Karelia Social and Health Care District, Lappeenranta, Finland

**Keywords:** Childhood brain tumor survivor, Radiotherapy, Disc degeneration

## Abstract

**Background:**

Childhood brain tumor (BT) survivors have an increased risk of treatment-related late effects, which can reduce health-related quality of life and increase morbidity. This study aimed to investigate lumbar disc degeneration in magnetic resonance imaging (MRI) in adult survivors of radiotherapy-treated childhood BT compared to age and sex-matched population controls.

**Methods:**

In this cross-sectional comparative study, 127 survivors were identified from hospital registries. After a mean follow-up of 20.7 years (range 5–33.1), 67 survivors (mean age 28.4, range 16.2–43.5) were investigated with MRI and compared to 75 sex-matched population-based controls. Evaluated MRI phenotypes included Pfirrmann grading, , intervertebral disc protrusions, extrusions, and high-intensity-zone-lesions (HIZ). Groups were also compared for known risk factors of lumbar intervertebral disc (IVD) degeneration.

**Results:**

Childhood BT survivors had higher Pfirrmann grades than controls at all lumbar levels (all *p* < 0.001). Lumbar disc protrusions at L4-5 (*p* = 0.02) and extrusions at L3-4 (*p* = 0.04), L4-5 (*p* = 0.004), and L5-S1 (*p* = 0.01) were significantly more common in the BT group compared to the control. The survivor cohort also had significantly more HIZ-lesons than the controls (n=13 and n=1, p=0.003). Age at diagnosis was associated with lower degree of IVD degeneration (*p* < 0.01). Blood pressure correlated with IVD degeneration (*P* < 0.05).

**Conclusions:**

Signs of early disc degeneration related to tumor treatment can be seen in the IVDs of survivors. Disc degeneration was more severe in children treated in adolescence.

## Introduction

Brain tumors (BT) are the most common solid tumors and the second most common malignancy after leukemia in childhood, with an annual incidence of 4–6/100 000 children [1–3]. The incidence is higher in North America and Europe [2]. The most common histological types of childhood BT are astrocytomas (40%) and medulloblastomas (20%) [2–4]. Childhood BT have an overall 78% 5-year survival rate [2, 3]. Over the last two decades, childhood BT survival has increased remarkably due to advances in molecular characterization of the tumors, improved surgery, chemotherapy, and radiation protocols [5, 6]. An increasing population of childhood BT survivors highlights the importance of understanding the long-term late effects of the BT and its treatment. Depending on the tumor’s histology and the patients’ age, treatment can be surgical excision, radiation, chemotherapy, or any combination of these. Radiotherapy can be delivered locally or to the whole brain, with or without spinal radiotherapy. Immature organ systems are prone to injury after radiotherapy, resulting in treatment protocols avoiding radiotherapy in the youngest patients. Thus, children younger than five are often initially treated with chemotherapy [7].

Radiotherapy-treated young adult survivors of childhood BT suffer similar late effects as can normally be found in the elderly population. These symptoms include hypertension, hearing impairment, diabetes, and cerebrovascular disease. This phenomenon has been recognized as early aging in the survivors [7, 8]. Low bone mineral density, spinal muscle hypotrophy, and disturbed growth of vertebrae potentially leading to scoliosis and kyphosis are other well-known late effects and signs of early aging in childhood cancer survivors [9–11]. These musculoskeletal late effects are reported in up to 80% of patients following childhood CNS radiotherapy with risk factors, including young age at initiation of treatment, increased radiation dose, and asymmetric dose distribution [10–13].

Lumbar intervertebral disc (IVD) degeneration results from gradual structural and biochemical changes associated with pain and disability [14]. IVD degeneration is multifaceted, strongly mediated by biomechanical alterations, nutritional compromise, and genetics. Several environmental and lifestyle factors are associated with IVD degeneration, such as physical loading, obesity, and smoking [14]. Previous studies have shown that IVD degeneration can be identified in childhood; however, the prevalence increases with age [15]. Soon after the phase of rapid physical growth, individuals with lumbar IVD have an increased risk of recurrent lower back pain; in adulthood, individuals are predisposed to long-term risk of recurrent pain [14–18]. Early IVD degeneration, if detected, may result in long-term recurrent pain in the survivors and further decrease their health-related quality of life.

Few studies have investigated radiotherapy’s long-term effects on childhood cancers concerning the adult lumbar spine. This study aimed to analyze the late-sequelae on the lumbar spine, focusing on the IVDs on radiotherapy-treated childhood BT survivors and comparing the findings to sex-matched population-based controls. This study is a part of a larger study of late effects on radiotherapy-treated childhood BTs. Previous studies have investigated vertebral late effects and health-related quality of life [19, 20].

## Methods

We investigated a national cohort of radiotherapy-treated childhood BT survivors diagnosed between 1970 and 2008, emphasizing lumbar IVD degeneration. Altogether, 127 survivors were identified from the registers of Oulu, Kuopio, Turku, Tampere, and Helsinki university hospitals using ICD-10 codes C70-72, D32-33 and D42-43. [8]. The inclusion criteria were 1) diagnosis of BT at the age of < 16 years, 2) radiotherapy as a part of their treatment, 3) age at the follow-up visit ≥ 16 years, 4) follow-up time since cessation of the tumor treatment ≥ 5 years, and 5) no known progressive BT at the time of the study. Information on BT and its treatment was gathered from the patient files. A medical physicist analyzed dose distributions using patients’ charts, treatment plans, and radiation field images to determine the radiation doses.

Of the 127 eligible survivors, 40 declined to participate in the study, and 13 were lost to follow-up. Among 74 survivors with consent, two did not undergo magnetic resonance imaging (MRI), due to claustrophobia or vagus nerve stimulator. MRI images were unavailable at the time of analysis for five participants. Altogether 67 survivors were included in the study (males *n* = 43) at a mean age of 28.4 years (range, 16.2–43.8). The mean age at diagnosis was 8.4 years (range, 1.1–15.7), the mean follow-up time was 20.7 years (range, 5–33.1). Half (52%) of the patients had an infratentorial tumor (*n* = 35). Glial cell tumors (*n* = 23) and embryonal tumors (*n* = 20) were the most common tumor types, followed by ependymomas (*n* = 9), germ cell tumors (*n* = 7), and other tumors (*n* = 4). In four patients, the histology remained unknown. Of the participants 58% (*n* = 39) received conventional radiotherapy with spinal radiotherapy and 42% (*n* = 28) conventional radiotherapy alone. The mean radiation dose given to the tumor bed was 51.2 Gy (range, 30–65.4). Altogether, 64% (*n* = 43) of the participants were treated with additional chemotherapy.

Clinical examination, laboratory tests, and spinal MRI were conducted during a two-day hospital visit. During the follow-up visit, information on blood pressure (BP), total cholesterol (S-kol), bone mineral density (BMD), body mass index (BMI), waist circumference, exercise level, lumbar MRI, and health-related quality of life (RAND-36 questionnaire) were gathered (Table 1). BMD was measured using dual X-ray absorptiometry (DXA), with Z-score ≤ -2.0 indicating ‘below the expected range for age and sex’ as recommended by the International Society for Clinical densitometry [21].

**Table 1:** Characteristics of survivors and controls

	**Survivor** Mean and [SD]	**Control** Mean and [SD]	***p*** **-value**
Number participants	**67**	**75**	
Age	**28.4** (range, 16.2–43.8) [6.9]	**29.2** (range, 29.0–30.0) [0.5]	0.29
Systolic BP	**130.5** (range, 101–167) [17.0]	**112.0** (range, 85–132) [11.2]	** < 0.001**
Diastolic BP	**80.8** (range, 54–107) [11.7]	**73.6** (range, 57–91) [7.0]	** < 0.001**
Total S-kol	**4.8** (range 3.1–6.8) [1.1]	**4.5** (range 2.8–6.0) [0.8]	0.21
BMI	**25.4** (range, 17.4–40.7) [6.7]	**23.2** (range, 16.7–34.2) [4.0]	**0.02**
Waist circumference	**90.4** (range, 67.6–142.6) [15.5]	**87.2** (range, 66.5–137) [12.8]	0.24
HI sports	**37%** (25/67)	**33.3%** (25/75)	0.81
Smoking	**13%** (9/67)	**16%** (12/75)	0.69
BMD ≤ 2	**6.7%** (10/67)	NA	NA

The differences between the survivors and controls are shown using the combination of means, range and standard deviations (SD) or percentages and counts for baseline characteristics. We tested the differences using t-tests and chi-squared tests. Age (years), *BP* Blood pressure (mmHg), *S-kol* Serum cholesterol (mmol/l), *BMD* Bone mineral density, *BMI* Body mass index (kg/m^2^), waist circumference (cm), *HI* High impact sport is defined as a minimum one-time, weekly exercise that causes sweating for 30 + minutes and smoking as regular smoking on a weekly basis

Findings of the survivors were compared to a sex-matched population control group (*n* = 75, males *n* = 45) (Table 1) randomly gathered from the Northern Finland Birth Cohort 1986 (NFBC1986) [22, 23]. NFBC1986 is a longitudinal birth cohort study with participants’ (*n* = 9479) expected date of birth between July 1, 1985, and June 30, 1986 (University of Oulu 1986) [22, 23]. The mean age of the control group was 29.2 years (range, 29.0–30.0), which did not differ from the mean age of the survivors (*p* = 0.29). All 75 control subjects underwent spinal MRI and clinical examination between 2015 and 2016.

### Spinal MRI methods and analysis

Spinal MRI was conducted using a Magnetom Espree 1.5 T scanner in Oulu, a Siemens Avanto 1.5 T scanner in Helsinki, Kuopio, and Tampere (Siemens, Erlangen, Germany), and a Philips Ingenia 1.5 T scanner in Turku (Philips Healthcare, Amsterdam, the Netherlands). A spinal MRI was performed after contrast-enhanced brain MRI and included T1-weighted turbo spin echo (TSE) sagittal and T2-weighted TSE sagittal sequences. The MRI images were analyzed by a musculoskeletal radiologist (R.K.).

**Fig. 1:**
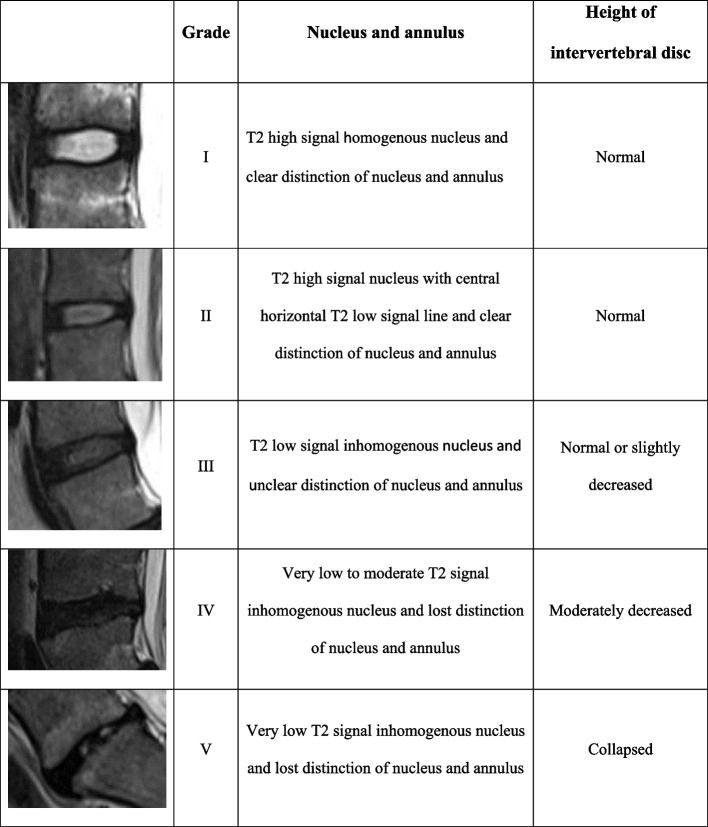
Pfirrmann classification of intervertebral disc degeneration, modified from Pfirrmann et. al [24]

**Fig. 2:**
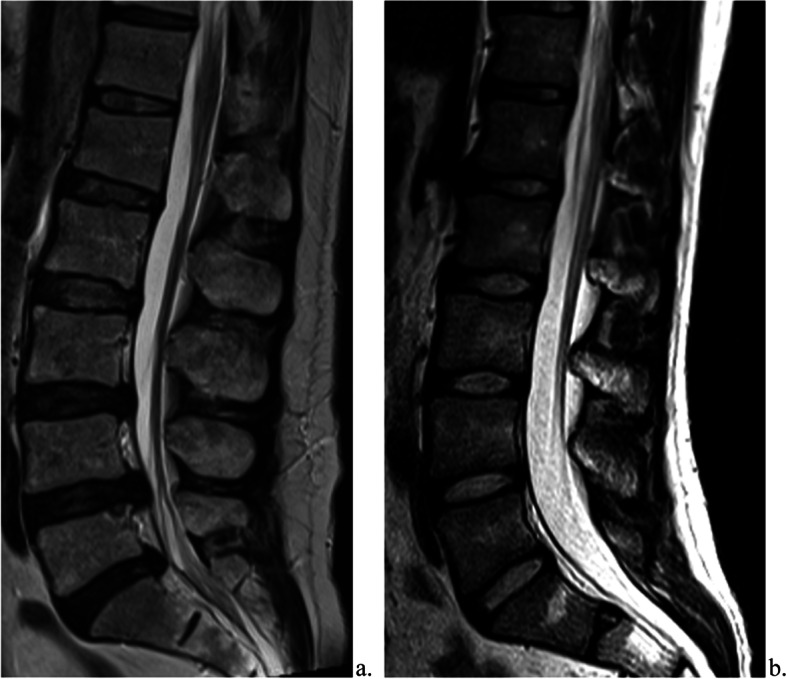
T2 weighted sagittal magnetic resonance image of one survivor (**a**) and one control (**b**)**.** The lumbar spine of a survivor (**a**) shows low T2 signal of the intervertebral discs L3-L4, L4-L5 and L5-S1 with lost distinction of nucleus and annulus (Pfirrmann IV). There is also extrusions of the forementioned intervertebral discs. The lumbar spine of a control (**b**) shows normal T2 signal of intervertebral discs without protrusions or extrusions

Lumbar IVD degeneration was graded from T2-weighted sagittal images using the Pfirrmann classification system (Fig. 1) [24]. Intervertebral disc extrusions, IVD protrusions, high-intensity zone (HIZ) lesions of IVD, spondylolysis / -listhesis and Schmorl’s nodes, were evaluated as defined in the literature (Fig. 2 a and b) [25]. Any additional abnormal changes in the IVDs and vertebraes were registered. Bone marrow edema was not evaluated due to lack of T2 fat-saturated sequences in the survivors.

### Statistical analysis

Differences in baseline characteristics and findings on MRI between patients and controls were calculated using t-tests and the chi-square test. Values are expressed in mean and standard deviation (SD). Further comparison was made using the Mann–Whitney U test to check for a correlation between the patient’s age at initiating treatment and Pfirrmann grades at the last follow-up, as well as the correlation between the follow-up time and final Pfirrmann grades. Patients were divided into low (< 3) and high (≥ 3) Pfirrmann-grade groups; the values were expressed using medians and interquartile ranges (IQRs). A *p*-value < 0.05 indicates a statistically significant difference. R v4.1.3 (R Foundation for Statistical Computing, Vienna, Austria) was used for statistical calculations.

## Results

**Fig. 3:**
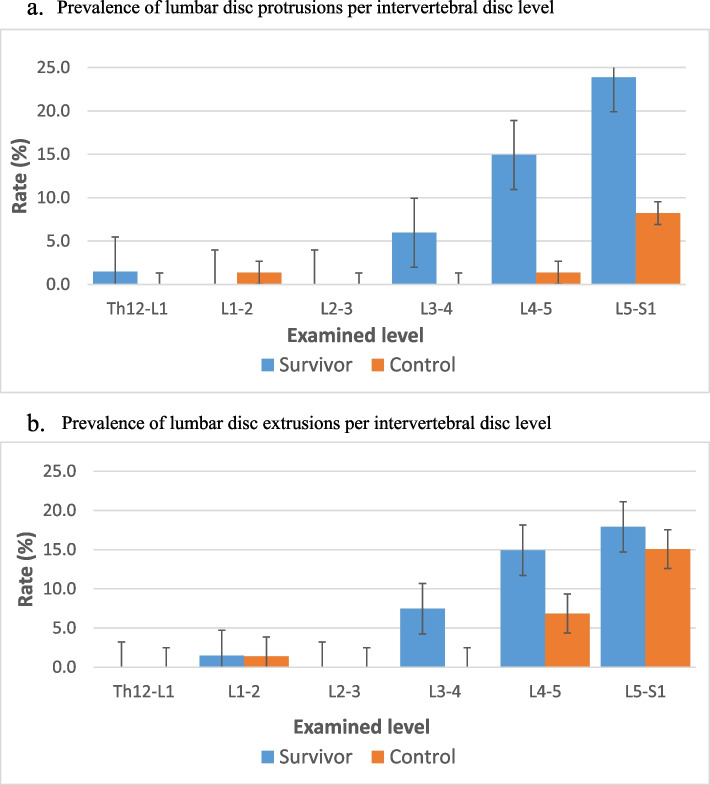
Prevalence of lumbar disc protrusions (**a**) and extrusions (**b**) between survivors and control subjects. The difference was statistically significant for protrusions at L4-5 (*p* = 0.02) and extrusions at L3-4 (*p* = 0.04), L4-5 (*p* = 0.004), and L5-S1 (*p* = 0.01)

The survivors had a higher prevalence of lumbar IVD degeneration both with respect to overall degeneration and degeneration at the individual disc levels in comparison to the control group (*p* < 0.001 for all) (Table 2). The survivors also had more IVD protrusions and extrusions than the control group, with a significant difference in the overall prevalence of disc herniations (*p* < 0.001) (Fig. 3a and b). When looking at specific IVD there was a statistically significant difference for extrusions on L3-4 (4 vs. 0, *p* = 0.04), L4-5 (10 vs.1, *p* = 0.004 and L5-S1 (16 vs. 6, *p* = 0.01) and protrusion on level L3-4 (5 vs. 0, *p* = 0.02). In addition, the survivor cohort had significantly more HIZ-lesions (*n* = 13) than the control (*n* = 1) (*p* = 0.003). All the HIZ-lesions affected the lower spine (L4-5 *n* = 8, L5-S1 *n* = 5). Spondylolisthesis (L5-S1) was found in two control subjects, while one survivor had spondylolysis at L4-5. No difference between the groups was found in the overall or per level prevalence of Schmorl’s nodes (*p* ≥ 0.05). Ten of the survivors had an annular tear (L3-4 *n* = 1, L4-5 *n* = 6, L5-S1 *n* = 2, L4-5 and L5-S1 *n* = 1), and an additional three patients had venous malformations of the vertebrae (L1 *n* = 1, L4 = n1, Th 8,9 and 11 *n* = 1). None of the control had annular tears nor venous malformations. However, one control patient had an L4 ring apophyseal injury. No difference was found between the survivor’s sex and Pfirrmann grade, protrusions, extrusions, or HIZ lesions (*p* > 0.05) between the forementioned radiotherapy treatment groups.

**Table 2:** Prevalence of intervertebral disc degeneration per level

	**Pfirrmann grade**		
Level	**Survivor** Mean and [SD]	**Control** Mean and [SD]	***p*** **-value**
Th12-L1	2.5 [0.6]	2.1 [0.4]	** < 0.001**
L1-2	2.6 [0.6]	2.1 [0.4]	** < 0.001**
L2-3	2.7 [0.6]	2.2 [0.5]	** < 0.001**
L3-4	2.9 [0.6]	2.3 [0.6]	** < 0.001**
L4-5	3.2 [0.7]	2.6 [0.9]	** < 0.001**
L5-S1	3.3 [0.8]	2.9 [0.9]	**0.01**
All levels	2.9 [0.3]	2.4 [0.3]	** < 0.001**

Prevalence of intervertebral disc degeneration (Pfirrmann grade) at individual levels and all levels combined among survivors and controls. T-test was used for comparison. *Th* Thoracic vertebrae, *L* Lumbar vertebrae, *S* Sacrum

Older age at BT diagnosis correlated with worse IVD degeneration at the follow-up visit (*p* < 0.01) (Table 3) and a higher BP correlated with a higher degree of IVD degeneration at levels Th12-L1 (*p* = 0.01), L1-2 (*p* = 0.01), and L3-4 (*p* = 0.03). No correlation was found between the degree of IVD degeneration and follow-up time, spinal radiation dose, location of radiation, chemotherapy, sex, participation in high or low-impact sports, smoking, BMD z-score ≤ -2.0, total cholesterol, BMI, or waist circumference (*p* > 0.05). Also, no correlation was noticed between Pfirrmann grade and RAND-36 full- or subscores (*p* > 0.05).

**Table 3:** Relationship between Pfirrmann grade and age

	**Pfirrmann < 3**	**Pfirrmann ≥ 3**	
Level	**Median age of survivors (IQR)**	**Median age of survivors (IQR)**	***p*** **-value**
Th12-L1	5.9 (4.4–11.3)	9.9 (6.0–12.2)	0.06
L1-2	5.8 (4.2–10.3)	10.3 (5.8–13.2)	**0.01**
L2-3	5.8 (3.3–11.0)	9.7 (5.6–12.7)	**0.04**
L3-4	5.3 (2.5–11.2)	8.7 (5.6–12.4)	0.08
L4-5	4.9 (2.5–10.8)	8.8 (5.5–12.2)	**0.05**
L5-S1	4.4 (1.9–5.8)	9.3 (5.6–12.2)	**0.01**

The relationship between survivors age at diagnosis and Pfirrmann grade at last follow-up. Analysis was done using the Mann–Whitney U test; results were expressed as the median and interquartile range (IQR). *Th* Thoracic vertebrae, *L* Lumbar vertebrae, *S* Sacrum

## Discussion

Our results indicate that lumbar IVD degeneration’s prevalence is higher in long-term survivors of radiotherapy-treated childhood BT survivors compared to sex-matched population-based controls of similar age. Survivors were more likely to have a higher degree of IVD degeneration at follow-up if the treatment was conducted in adolescence.

Early aging in childhood tumor survivors has been described [7, 8]. Lumbar IVDs show degenerative changes during normal aging, increasing from 37% among 20-year-olds to 96% among 80-year-olds [25]. In the current study, we observed a significantly higher prevalence of IVD degeneration in long-term survivors of radiotherapy-treated childhood BT, implicating that cancer treatment-related early aging also affects IVD. IVD degeneration is well-known to precede other lumbar degenerative changes, such as disc herniations, spinal stenosis, and Modic changes [14]. Survivors had an increased prevalence of lumbar IVD herniations, but further studies are required to better understand the long-term consequences of IVD in this population. Lumbar IVD degeneration is associated with low back pain [16, 17]. A previous publication of the same BT survivor population showed a trend toward reporting increased pain compared to healthy controls, but this association did not reach significance [20]. Our results suggest that long-term BT survivors may be at risk for low back pain due to IVD degeneration later in life.

We observed significantly more HIZ-lesions in BT survivors. An association of HIZ-lesions with low back pain has been suggested [26], but the topic remains controversial. Annular fissures may further increase the likelihood of developing IVD degeneration [27]; longitudinal follow-up will show whether this hypothesis holds true.

IVD degeneration’s etiology is multifaceted; no single key contributor to the IVD degeneration process exists [14]. The importance of heredity has become evident through twin studies, with genetic factors explaining over 70% of phenotypic variation in IVD degeneration [28]. Nutrition of avascular IVD is supplied through a few blood vessels entering the annulus fibrosus and bone-cartilage endplate junction [29]. Atherosclerotic risk factors, such as obesity and smoking, have been associated with IVD degeneration [30–32]. Atherosclerosis is common in BT survivors, partly due to radiotherapy’s direct effects on the vessel walls [32, 33]. General atherosclerosis may thus be one factor leading to IVD degeneration in these patients. This has implications for preventive interventions to reduce risk of IVD degeneration, as focusing to reduce the risk of overweight, smoking, and other atherosclerotic risk factors may have beneficial effects in BT survivors especially when they are treated in adolescence.

Radiotherapy is standard care in malignant childhood BT tumors. There is a lack of studies regarding the incidence and severity of IVD degeneration in childhood tumor survivors; to our knowledge, this is the first publication to describe the prevalence of IVD degenerative changes in this patient cohort. We believe no publications describe CNS radiation- and chemotherapy related IVD degeneration in humans. In animal models, genotoxic stress has accelerated age-associated degenerative changes in IVDs [34]. The hypothesis is that oxidative stress of radiation- and chemotherapy might lead to IVD degeneration. However, this study does not establish a causal relationship between tumor treatment and IVD degeneration.

We observed that age at treatment significantly correlated with the degree of IVD degeneration and that treatment conducted at a younger age yielded a substantially lower degree of IVD. This is in contrasts to other studies which have found no relationship between the age of treatment and the severity of early aging [7]. Younger children have a greater regeneration potential, which can be seen in diseases such as Perthes, where younger age at presentation leads to better outcomes in adulthood [35]. In IVD, notochordal cells with regenerative potential are slowly replaced with mesenchymal cells, and notochordal cells are absent by age 10 [36]. Selection of treatment modality may have different consequences on long-term spinal health depending on growth phase during cancer treatment and should be considered in future studies.

This study’s strengths are a long-term follow-up, the use of validated methods of grading IVD degeneration, systematic MRI screening, a high participation rate, and comparison to population-based controls. Limitations of this study include heterogeneous tumor histology and lack of longitudinal follow-up to observe the incidence of IVD degeneration. Additionally, these patients were treated with different chemotherapy protocols and radiotherapy techniques, although the total radiation dose administered to CNS was relatively homogenous.

## Conclusions

This study shows that IVD degeneration is common in radiotherapy-treated childhood brain tumor survivors, with a higher degree of severity in children treated during adolescence.

## Data Availability

The datasets generated and/or analyzed during the current study are not publicly available due to their size and complexity but are available from the corresponding author upon reasonable request.

## Abbreviations

BMD: Bone mineral density
BMI: Body mass index
BP: Blood pressure
BT: Brain tumor
CNS: Central nervous system
DXA: Dual X-ray absorptiometry
HIZ: High-intensity-zone lesion
IVD: Intervertebral disc
IQR: Interquartile range
MRI: Magnetic resonance imaging
SD: Standard deviation
S-kol: Total serum cholesterol
TSE: Turbo spin echo
